# Stress modulates gastric interoception depending on eating traits and emotion regulation: evidence from the magic table

**DOI:** 10.1038/s41598-026-48641-w

**Published:** 2026-05-13

**Authors:** Miriam Kipping, André Schulz, Olga Pollatos

**Affiliations:** 1 Clinical and Health Psychology, Institute of Psychology and Education, Ulm University, Albert-Einstein-Allee 45, 89081 Ulm, Germany; 2 Department of Behavioural and Cognitive Sciences, Faculty of Humanities, Education and Social Sciences, University of Luxembourg, 11, Porte des Sciences, Esch-sur-Alzette, 4366 Luxembourg

**Keywords:** Satiation perception, Gastric interoception, Eating, Stress, Emotion regulation, Gastroenterology, Health care, Physiology, Psychology, Psychology

## Abstract

The high prevalence of obesity and low treatment response in eating disorders highlight the need for a better understanding of eating regulation. Gastric interoception – the perception of signals such as hunger and satiation – plays a key role in aligning food intake with physiological needs. This study investigated how acute stress, emotion regulation difficulties, and eating traits influence gastric interoceptive sensitivity. Ninety-four fasting participants completed both a stress and control task in counterbalanced order across two laboratory sessions. Gastric interoceptive sensitivity was assessed using a novel method, the Magic Table (MT), in which participants ate yogurt from a self-refilling bowl until satiation and fullness. Multi-level modeling evaluated stress effects and moderation by self-reported eating traits and emotion regulation. For validation, 61 participants also completed the established Two-step Water Load Test (WLT-II) and free food consumption. Medium to strong correlations between MT indices, WLT-II, and food intake supported the validity of the MT. While stress had no overall effect on gastric interoception, cross-level interactions showed reduced fullness sensitivity under stress in individuals with high emotion regulation difficulties, restrained, and uncontrolled eating. These findings suggest that this group may be at risk for interoceptive desensitization under stress and inform studies on interoception-focused interventions.

**Supplementary Information:**

The online version contains supplementary material available at 10.1038/s41598-026-48641-w.

## Introduction

The prevalence of obesity is increasing globally^1^. In 2022, over 16% of adults worldwide were living with obesity, and more than 43% were overweight^2, 3^. This trend is alarming given the long-term consequences, such as type 2 diabetes and arteriosclerosis, which contribute to disability, reduced life expectancy, and diminished quality of life^2, 4^. Additionally, eating disorders often co-occur with obesity or underweight, are linked to somatic and mental health comorbidities, reduced quality of life, and higher mortality and suicide risk compared to the general population^5, 6^. Available treatments show only low to moderate response rates^7, 8^, underlining the need for a better understanding of the mechanisms underlying eating behavior to develop more effective prevention or treatment approaches.

Stress is a key factor in food intake regulation. Physiologically, acute stress triggers the sympathetic adrenal medullary (SAM) axis, which supports energy mobilization for a fight-or-flight response and inhibits behaviors such as eating^9^. However, empirical findings are mixed: experimental and correlational studies and a meta-analysis show that stress in particular increases the consumption of sweet or high-fat foods^10–20^. In contrast, a recent study and two meta-analyses reported no or only minimal changes in eating behavior under stress or negative emotions^21, 22–23^. These inconsistencies may stem from between-person differences. For example, among female students, 19% reported no change in appetite under stress, 51% reported an increase, and 30% a decrease^24^. Such variability has been linked to individual traits, life stress, and physiological stress responses^12, 25–33^. One study found that high emotional eaters reported less reduction in hunger after eating under stress than low emotional eaters, suggesting reduced gastric interoception under stress^34^. Yet, no study has directly assessed whether stress alters gastric interoceptive sensitivity (a typical behavioral indicator of interoception in the gastric domain, assessing the perception threshold of satiation). While interoception is often studied as a stable trait, little research has examined it under different psychophysiological states, like stress. However, in the cardiac modality, some evidence suggests that acute stress affects interoceptive accuracy (i.e., the correct perception of one’s heartbeat^35^, possibly due to stronger organ signaling and neural representation^36–39^. These findings raise the possibility that stress might also alter gastric interoception and thereby influence stress-related eating.

Research on interoception and dysregulated eating showed reduced interoception in overweight and obese individuals, as well as in patients with eating disorders like anorexia nervosa, bulimia nervosa, and binge eating disorder^40–43^. Eating disorders are severe mental health conditions characterized by persistent and clinically significant disturbances in eating behavior (e.g., severe dietary restriction or recurrent binge eating episodes), body image, and weight regulation that cause substantial physical and psychosocial impairment^44^. In contrast, in healthy individuals, elevated BMIs, binge eating, and emotional, uncontrolled, or restrictive eating, and emotional regulation difficulties can be observed as subclinical traits. Interoceptive deficits are also linked to these dysfunctional traits (e.g., higher body mass index (BMI), binge eating, emotional eating, and emotion regulation difficulties) in subclinical and healthy samples^45–50^. Although eating disorders are qualitatively distinct conditions from dysfunctional eating traits and emotion regulation difficulties, these traits were identified as risk factors for or symptoms of eating disorders^44, 51, 52^. Therefore, examining these traits in a healthy sample, along with interoception, allows investigation of the mechanisms that may contribute to dysregulated eating. So far, research has linked dysregulated eating mainly to cardiac interoceptive accuracy or general interoceptive sensibility (i.e., bodily perceptions assessed with self-report questionnaires)^53^. However, gastric interoception is likely more directly relevant to eating behavior regulation^54^. For instance, hunger/satiety-specific interoceptive sensibility has been found to relate more strongly to disordered eating symptoms than general interoceptive sensibility^55^.

Few studies have examined gastric interoception using behavioral methods. One reported increased gastric interoceptive sensitivity (i.e., drinking less water until fullness) in women with anorexia nervosa^56^, while others found reduced sensitivity (i.e., drinking more until satiation and fullness perception) for patients with bulimia nervosa and binge eating disorder as compared to healthy individuals^57^. These used the one- or two-step Water Load Test (WLT-II). In the WLT-II, participants drink water in two stages: until satiation and then until fullness to assess gastric interoceptive sensitivity^58, 59^. The WLT-II offers the opportunity to investigate the perception of satiation through gastric distension. Additionally, hormone release and taste influence hunger and satiation^60^. To generalize findings to real eating behavior, it therefore appears important to assess gastric interoceptive sensitivity in a food-based context. In the present study, we used the Magic Table (MT), a new method adapted from the WLT-II procedure^59^, to assess gastric interoceptive sensitivity in a setting involving food intake.

Within the interoceptive framework, gastric interoceptive sensitivity refers to the behavioral detection threshold at which gastric sensations become consciously perceivable^58, 59^. In the MT, this is operationalized as two perceptual thresholds during gastric filling with food: satiation, reflecting the perception of an early, relatively weak gastric signal, and fullness, reflecting the perception of a stronger and more salient signal. These sensations arise from gastric distension and nutrient-related signaling, detected by mechanosensors and chemoreceptors in the gastrointestinal tract, and are communicated to the brain via afferent neural and endocrine pathways^61^. Physiological characteristics of organs (e.g., stomach capacity, receptor sensitivity, or cardiac contractility) contribute to the generation of interoceptive signals and therefore inevitably influence their perceptibility. Consequently, interoceptive measures, whether gastric, cardiac, or self-report, reflect both the individual’s bodily signals and the perception of these signals. Unlike exteroceptive tasks, in which stimuli can be standardized, interoception inherently involves the perception of physiology that varies from person to person. Importantly, behavioral measures of interoceptive sensitivity, accuracy, and sensibility capture the interaction between physiological signal generation and perceptual processes rather than the properties of the organ alone. Within the broader interoceptive framework, however, the processing of bodily signals does not occur in isolation but is embedded in a multisystem integration of afferent information^62^. Alongside visceral inputs, additional influences, such as the hedonic value of food, prior experience, motivational states, and contextual factors, can enter the processing stream both centrally and peripherally and shape how interoceptive signals are weighted, interpreted, and brought to awareness. Thus, gastric interoceptive sensitivity, as defined and operationalized here, can be distinguished conceptually from other regulatory processes such as reward-driven eating and cognitive control, while acknowledging that these processes may converge at the level of central integration and modulate the subjective experience of gastric sensations^61, 63^.

In summary, evidence suggests that both stress and interoception influence eating behavior and that this influence varies depending on between-person differences in eating habits and emotion regulation abilities. However, no study has yet investigated whether stress-induced changes in gastric interoceptive sensitivity differ between individuals – that is, whether stress sensitizes or desensitizes gastric interoceptive sensitivity depending on individual characteristics. In this study, we used a new behavioral approach involving food intake to assess gastric interoceptive sensitivity in high- and low-stress conditions. Our aims were:

1. To validate the MT against the established WLT-II and free food consumption, expecting medium to strong correlations.
2. As a manipulation check, to confirm higher self-reported stress after stress induction compared to a control condition.
3. To exploratively examine whether stress affects gastric interoceptive sensitivity.
4. To test whether individuals with higher levels of restrained, uncontrolled, emotional eating, or more emotion regulation difficulties show reduced gastric interoceptive sensitivity under stress compared to a control condition.

## Methods

### Participants

The sample size needed to detect an interaction effect with a repeated measures analysis of variance (RM-ANOVA) was determined using G*Power^64^, assuming a medium effect size (*f* = 0.30). This was used as a proxy for our multilevel models (MLMs) as power was shown to be similar or higher for MLMs compared to RM-ANOVAs^65, 66^. The estimated sample size to detect a significant effect (*α* = 0.05) with 80% power was *N* = 90. We slightly over-recruited the study to compensate for potential dropout or technical assessment problems. *N* = 94 healthy participants (81.9% females) between 19 and 33 years of age (*M* = 22.61, *SD* = 2.85) were recruited at a university in southern Germany. Exclusion criteria were mental, neurological, or endocrinological disorders, and regular use of medication (except for contraceptives). One participant did not participate in the second laboratory appointment. Their data from the online questionnaire and first session were retained in the multilevel models. All participants gave their written informed consent after receiving a description of the experimental procedure before the start of the study. Their participation was voluntary, and they received either course credit or money as compensation for their involvement. The entire research protocol adhered to the principles outlined in the Declaration of Helsinki and received formal approval from the ethics committee of Ulm University [reference number: 168/23, 06/07/23].

### Procedure

**Fig. 1:**
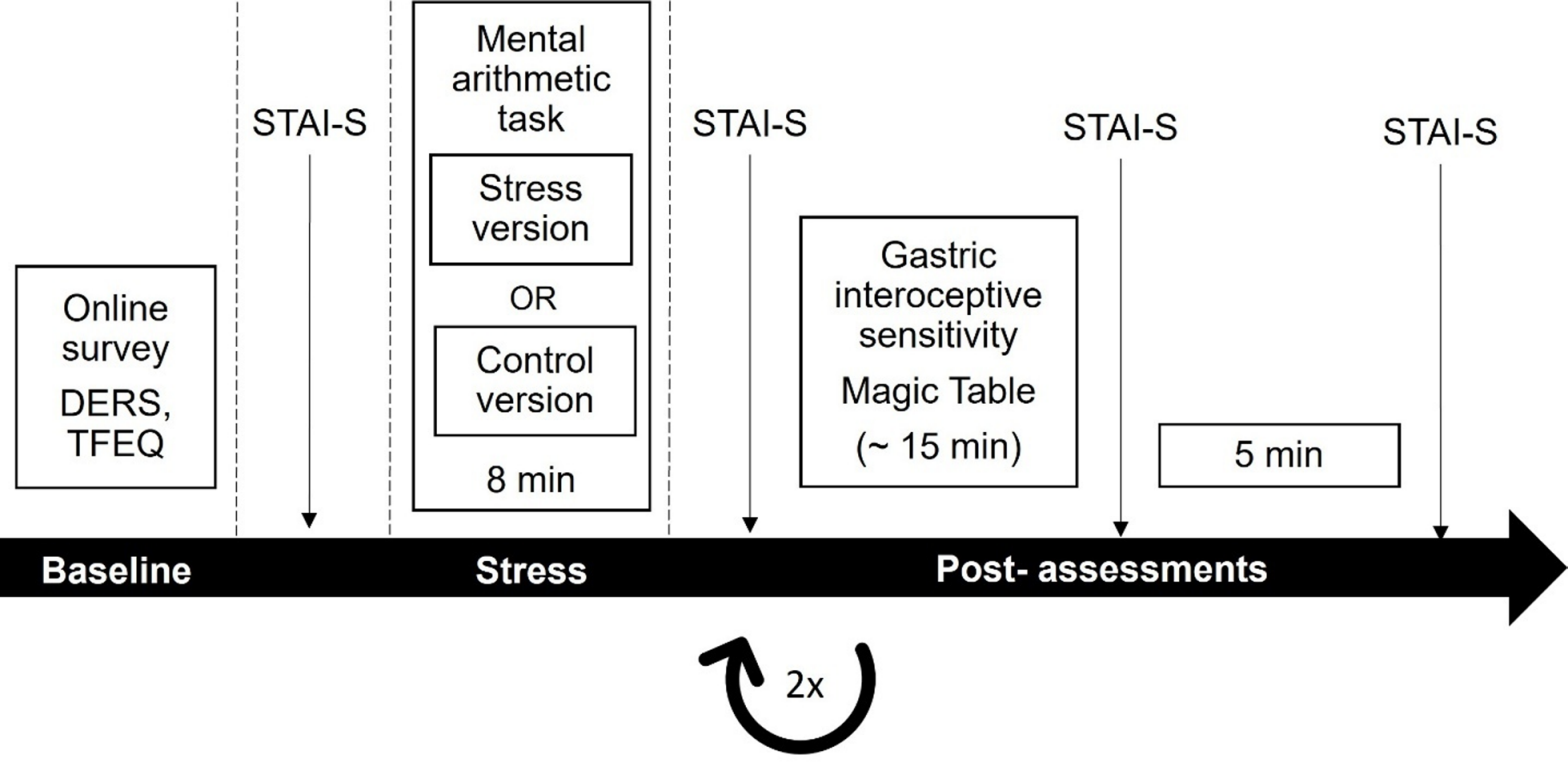
Study procedure. DERS, Difficulties in Emotion Regulation Scale; TFEQ, Three-Factor-Eating-Questionnaire; STAI-S, State scale of the State-Trait Anxiety Inventory.

The study consisted of three parts: an online questionnaire and two laboratory sessions (see Fig. 1 for the study procedure). First, participants answered the online questionnaire, which included demographic information, the Three-Factor-Eating-Questionnaire (TFEQ), and the Difficulties in Emotion Regulation Scale (DERS).

In a single-blind repeated measures cross-over design, we assessed the gastric interoceptive sensitivity of all participants using the MT once in the stress and once in the control condition in a balanced order across both laboratory sessions. Laboratory sessions were separated by at least 48 h to minimize carry-over effects. Participants were told that the study aims to assess the interactions between hunger, cognitive performance, and emotional state. They did not receive any specific information regarding the purpose of the MT.

In both laboratory sessions, participants arrived at the laboratory between 8 a.m. and 11 a.m. after fasting for 8 h. Both laboratory sessions took place at the same time for each participant. In the first session, participants were queried by the experimenter about exclusion criteria and received written and oral information on the study procedure. In addition, their weight was measured in kilograms and their height in meters to one decimal place. The following procedure was identical for both laboratory sessions: Participants completed the state scale of the State-Trait Anxiety Inventory (STAI-S) to measure baseline affect. Thereafter, they completed the stress or control version of the mental arithmetic task. Immediately afterwards, they again rated the STAI-S. We then measured gastric interoceptive sensitivity with the MT paradigm. Finally, participants again completed the STAI-S, which was repeated after 5 min of relaxation.

The sample size needed to validate the assessment of satiation perception with the WLT-II and free food consumption was determined using G*Power^64^ based on a medium effect size of (*r* = .30). The estimated sample size to detect a one-sided significant correlation (*α* = 0.05) with 80% power was 64. A subsample of *N* = 61 participants could be re-recruited at the end of the second laboratory session using the MT for a third and fourth laboratory session. They were informed that there was a second part of the study, this time on thirst, cognitive performance, and emotional state. For those who agreed to participate, the debriefing took place after all four laboratory sessions. Again, a randomized cross-over design was employed. The procedure was identical to that of laboratory sessions one and two, with the exception that the WLT-II was used in place of the MT. Following the WLT-II, participants watched a 10-minute relaxing landscape video to normalize emotional states. Subsequently, participants were offered a standardized breakfast for free consumption. After breakfast, either a second stress induction or a control task was administered, followed by an assessment of snack consumption during a waiting period. Results on stress-induced snack consumption are not presented here, as they fall outside the scope of the current research questions.

### Material design

#### Stress induction

Stress was induced utilizing a mental arithmetic test. We adopted the computer version of the Paced Auditory Serial Addition Test (PASAT-C^67^). Participants had the task of adding single-digit numbers (1–9), which were presented serially on a computer screen as correctly and quickly as possible. The response was collected using a keyboard. If it was incorrect or too slow (not within the inter-stimulus interval), a loud aversive tone sounded. After five training trials, the task lasted a total of 8 min, with gradually increasing difficulty after 2 min and 6 min. This was implemented via shorter inter-stimulus intervals (3 s, 2 s, 1.5 s). The control task was comparable, with the differences that the inter-stimulus interval (3 s) was not shortened and the numbers to be added were between 1 and 5. To increase induced stress, participants were told that the task assesses their cognitive performance.

#### The Magic Table to assess gastric interoceptive sensitivity

**Fig. 2:**
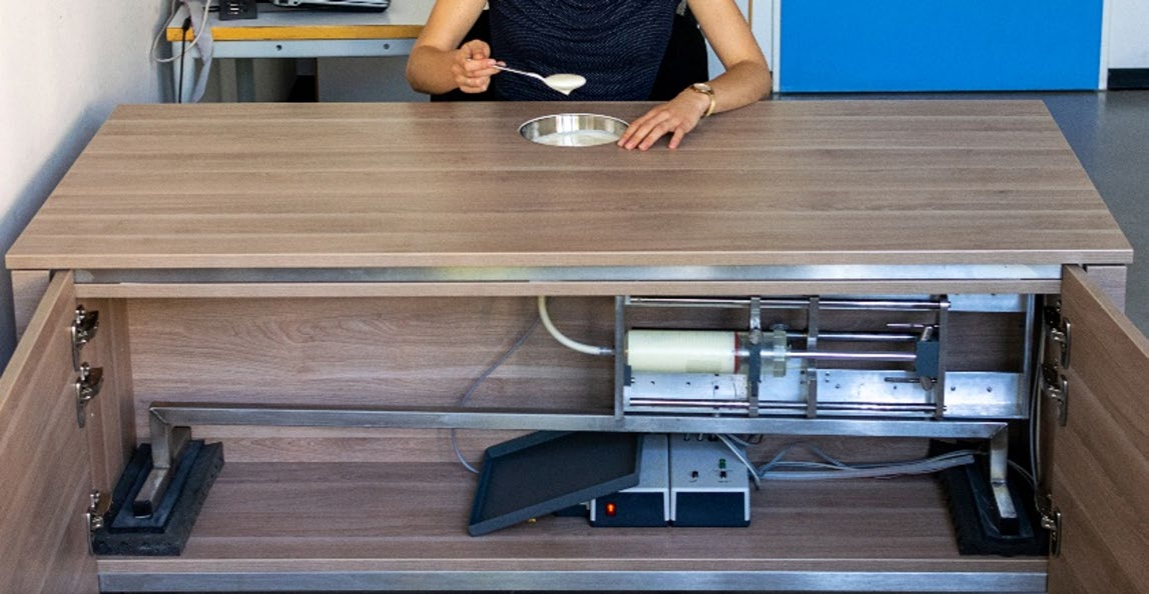
Depiction of the Magic Table. While participants are eating a hidden scale under the bowl and a hidden syringe and pump inside the table maintain a constant amount of yogurt inside the bowl.

The perception of satiation was assessed through food intake (yogurt) using the MT (see Fig. 2, and the Supplementary Material for more information; set-up similar to^68^). This method was developed according to the WLT-II^59^ for measuring gastric interoceptive sensitivity. Participants ate yogurt from a bowl using a customized table with a hidden scale underneath the bowl and a pump that refilled the bowl through an automatic mechanism to maintain a constant amount of yogurt in the bowl. This way, visual feedback on the amount of food eaten was minimized, ensuring that participants relied on internal signals of satiation. Following the WLT-II, participants were first instructed to eat until they experienced an initial feeling of satiation (sat_g) and second, until feeling completely satiated (Δfull_g). The experimenter measured the amount of yogurt eaten until both thresholds after the experiment. Since the capacity of the syringe, from which the yogurt was pumped into the bowl, was limited (500 g), an experimenter had to be present to replace almost empty syringes. Including nutrient-rich foods in the assessment of gastric interoceptive sensitivity broadens available satiation signals beyond gastric distension, as is the case in the WLT-II. In this study, participants could choose between three flavors of yogurt (vanilla (99 kcal / 100 g), raspberry (99 kcal / 100 g), and vegan blueberry (83 kcal / 100 g)) to consider the preference for sweet foods under stress. They ate the chosen flavor in both laboratory sessions.

Since gastric interoceptive sensitivity was assessed as a state variable in this study, we used the absolute amount of yogurt consumed until first satiation (sat_g) and the total amount consumed until fullness (total_g = sat_g + Δfull_g) as our primary measures. We did not use the sensitivity index (sat_%) (as described for trait-like assessments with the WLT-II^59^) to examine the effect of stress on gastric interoception because this index carries interpretive ambiguity in our repeated-measures design: a change in sat_% may reflect a change in sat_g, a change in total_g, or a change in both thresholds. Conversely, sat_% would remain unchanged if sat_g and total_g shift proportionally under stress, despite meaningful alterations in both absolute thresholds. Since these underlying mechanisms cannot be identified when analyzing changes in sat_%, we decided in this study to examine sat_g and total_g as direct indices of changes in gastric interoceptive sensitivity thresholds in response to stress. Moreover, the original rationale for using sat_% was to control for individual differences in stomach capacity^59^, a factor already accounted for by our within-subject design. Nevertheless, we use all indices described for the WLT-II to validate the MT with the WLT-II and free food consumption.

#### The WLT-II

Participants were asked to drink noncarbonated water in the first period until perceiving satiation and in the second period until reaching maximum stomach fullness. Each drinking period lasted for 5 min. Participants drank with a straw from nontransparent 5 l flasks filled with 1.5 l of water for safety reasons. This way, participants were blinded regarding the amount of water they drank and had to rely on internal cues for satiation and fullness. Absolute amounts of water ingested in grams until satiation (sat_g) and in total (total_g), as well as Δfull_g, and sat_% were used as measures of gastric interoceptive sensitivity (for a detailed description see^59^.

#### Free food consumption

Participants were offered a buttered bread (15 g butter per slice of bread) breakfast providing 20% of their daily energy requirement, consistent with studies showing that breakfast typically accounts for 16–21% of daily intake^30–32^. Energy requirements were calculated using the Harris and Benedict equation with an assumed activity index of 1.7^69^. Participants were informed they would receive breakfast to avoid prolonged fasting and were instructed to help themselves freely. The amount of buttered bread consumed in grams was calculated as the difference in weight of the plate before and after consumption. This task was intended to minimize the effects of fasting on subsequent snack consumption (reported elsewhere) and is used here to provide preliminary insights into the predictive validity of the MT indices.

#### Self-report measures

We used the German version of the STAI-S^70, 71^ to assess state anxiety in reaction to the stress induction with 20 items (e.g., “I am tense”) on a 4-point scale (1 = *not at all* to 4 = *very much*). The STAI-S has sound psychometric properties with a mean internal consistency of α = 0.91 (ω’s = 0.88 − 0.95 in the current sample) and a mean test-retest reliability of *r* = .70^72^.

Eating behavior traits were assessed with the German version of the TFEQ^73^ in its revised form^74^ with the subscales *restrained eating* (15 items, e.g., “I consciously hold back on eating so as not to put on weight”), *uncontrolled eating* (11 items, e.g., “Once I’ve started eating, sometimes I can’t stop”), and *emotional eating* (3 items, e.g., “When I’m anxious or tense, I often start eating”). The scales showed good internal consistency (restrained eating: α = 0.84 (here ω = 0.84); uncontrolled eating: α = 0.80 (here ω = 0.78); emotional eating: α = 0.78 (here ω = 0.71) and were positively associated with BMI^74^.

The German version of the DERS^75, 76^ was used to assess emotion regulation difficulties with 36 items (e.g., “When I have negative feelings, I get out of control”) on a 5-point scale (1 = *almost never [0–10%]*, 2 = *sometimes [11–35%]*, 3 = *about half the time [36–65%]*, 4 = *most of the time [66–90%]*, and 5 = *almost always [91–100%]*). It has a high internal consistency of α = 0.95 (ω = 0.95 in the current sample), with supporting evidence for convergent validity^76^.

### Data analysis

All data analyses were performed using RStudio (v4.4.1^77^) including the packages *ggpubr*^78^, *sjPlot*^79^, *psych*^80^, *rstatix*^81^, and *correlation*^82^. This study was not formally preregistered. However, hypotheses and the data-analytic plan were specified before the data were collected. *P*-values smaller than 0.05 were considered significant.

We computed Pearson correlations between the gastric interoception indices measured with the MT and WLT-II in the different stress states, as well as between the MT and free food consumption to validate the MT as a new method to assess gastric interoceptive sensitivity. Holm correction was applied to correct for multiple comparisons.

STAI-state scores were analyzed using constrained MLMs (cMLMs) with *Time* (t0, t1, t2, t3) and *Time* × *Condition* as fixed effects and participant as a random effect. In this model, baseline (t0) was included as part of the longitudinal outcome while baseline differences between conditions were constrained to zero, which is recommended for the analysis of repeated pre–post measurements in intervention studies^83, 84^. Significance of fixed effects was assessed using Type III *F*-tests with Satterthwaite approximation of degrees of freedom. Planned contrasts were computed using the *emmeans* package^85^ to test (1) within-condition changes from baseline to post-stress induction (t1 − t0) separately for each condition, and (2) whether this change was significantly larger in the stress than in the control condition, operationalized as (Stress_t1 − Stress_t0) − (Control_t1 − Control_t0). Effect sizes are reported as Cohen’s *d*_z_ for within-condition contrasts and Cohen’s *d*_av_ for the interaction contrast^86^.

Intraclass correlation coefficients (ICCs) indicated the hierarchical nature of the data for both satiation thresholds (sat_g: ICC = 0.83, total_g: ICC = 0.89)^87^. Therefore, MLMs were used to evaluate the effect of condition on gastric interoceptive sensitivity. MLMs, including the cross-level interactions of each trait with the fixed factor Condition, were used to test the hypothesized effect of stress as a function of emotion regulation difficulties and eating traits. The indices sat_g and total_g were used as dependent variables in the MLMs (for the rationale, see section “The Magic Table”). The MLMs included the traits of *Restrained*, *Uncontrolled*, *Emotional eating*, or *Emotion regulation difficulties* as second-level predictors. In all MLMs, we controlled for *BMI*, *Sex*, *Baseline stress* (STAI-S scores at t0), and *Stress reactivity*. Stress reactivity scores were computed by subtracting STAI-S scores at t0 from STAI-S scores at t1 for each condition. Baseline stress scores, stress reactivity scores, and person-level predictors (BMI, eating traits, emotion regulation difficulties) were grand mean standardized. Participants’ intercepts were modeled as random effects. MLMs were built with the package *lme4*^88^ using restricted maximum likelihood estimation and a random intercept. The package *performance*^89^ was used to check model assumptions and model outliers using plots. Influential outliers were defined based on Cook’s Distance > 0.9^90^. Three to six outliers were detected for the different MLMs. As deviations from the normality assumption for residuals and from the homogeneity of variances were also detected, tests of model parameters were performed with nonparametric residual bootstraps with 5000 bootstrap samples using percentile confidence intervals with the package *lmeresampler*^91^. The nonparametric residual bootstrap can deal with biases in the variance estimates and standard errors in the case of non-normal residuals^92, 93^.

As crossover designs are susceptible to period and carry-over effects^94^, two additional control analyses were carried out. A period effect was examined by including session (first vs. second laboratory visit) as a fixed effect in the MLMs on sat_g and total_g. A carry-over effect was examined by testing the interaction between session order and condition. Neither the period effect nor the carry-over effect reached statistical significance (both *p* > .05).

## Results

### Demographic and questionnaire data

Sample characteristics, including questionnaire data on eating behavior, emotion regulation, and STAI-S scores, are presented in Table 1. 74.5% of participants had a high school degree, and 25.5% had a university degree. The mean BMI of 22.2 indicates a predominantly healthy-weight sample. On average, low to moderate levels of uncontrolled, restrained, and emotional eating were reported, and emotion dysregulation was within the range expected for a non-clinical sample. Baseline STAI-S scores were comparable across conditions and sessions.

**Table 1:** Descriptive statistics of questionnaire data.

Variable	*M*	*SD*	Min	Max
BMI	22.20	2.68	16.71	30.33
TFEQ restrained eating	5.14	3.33	0	13
TFEQ uncontrolled eating	4.69	2.62	0	11
TFEQ emotional eating	0.72	1.01	0	3
DERS	69.32	18.29	39	119
STAI-MT sessions				
Control condition				
t0	36.30	7.23	24	57
t1	39.30	8.46	24	64
t2	35.60	6.76	22	56
t3	33.00	5.93	22	52
Stress condition				
t0	35.70	7.22	21	58
t1	46.90	10.50	27	69
t2	38.10	7.66	21	59
t3	34.60	6.72	23	54
STAI-WLT-II sessions				
Control condition				
t0	37.30	8.73	21	62
t1	38.90	9.07	21	68
t2	36.80	7.27	21	61
t3	35.00	8.35	23	73
Stress condition				
t0	37.30	8.32	21	60
t1	45.90	10.10	22	65
t2	38.30	6.81	22	55
t3	35.00	7.44	22	59

BMI, Body mass index; TFEQ, Three-Factor-Eating-Questionnaire; DERS, Difficulties in Emotion Regulation Scale; STAI, State-Trait Anxiety Inventory (state subscale); WLT-II, two-step Water Load Test; MT, Magic Table. Magic Table session: *n* = 93 (Control), *n* = 94 (Stress). WLT session: *n* = 60 per condition.

### Validation of MT with WLT-II and food consumption

The correlations between the MT and WLT-II in different stress states, as well as the MT in the control condition and the free food consumption, can be found in Table 2. Correlations for total_g and sat_g were significant, with strong effect sizes, while correlations for Δfull_g and sat_% showed small to medium effect sizes^95^. All indices of the MT in the control condition, except for sat_%, were highly correlated with the amount of buttered bread eaten in the free food consumption.

**Table 2:** Correlations between indices of interoceptive sensitivity of the MT, WLT-II, and free food consumption.

Index MT	Index WLT-II	*r*	*t*	df	*p*	95%-CI
total_g control	total_g control	0.50	4.48	59	< 0.001***	[0.29, 0.67]
total_g stress	total_g stress	0.56	5.18	59	< 0.001***	[0.36, 0.71]
sat_g control	sat_g control	0.55	5.12	59	< 0.001***	[0.35, 0.71]
sat_g stress	sat_g stress	0.53	4.79	59	< 0.001***	[0.32, 0.69]
Δfull_g control	Δfull_g control	0.37	3.08	59	0.012*	[0.13, 0.57]
Δfull_g stress	Δfull_g stress	0.41	3.48	59	0.005**	[0.18, 0.60]
sat_% control	sat_% control	0.35	2.83	59	0.019*	[0.10, 0.55]
sat_% stress	sat_% stress	0.18	1.42	59	0.301	[– 0.07, 0.41]

Index MT	Food consumption	*r*	*t*	df	*p*	95%-CI
total_g control	bread_g	0.61	5.85	59	< 0.001***	[0.42, 0.74]
sat_g control	bread_g	0.59	5.67	59	< 0.001***	[0.40, 0.74]
Δfull_g control	bread_g	0.46	3.93	59	0.001**	[0.23, 0.63]
sat_% control	bread_g	0.19	1.46	59	0.301	[– 0.07, 0.42]

MT, Magic Table; WLT-II, two-step Water Load Test; sat_g, amount of yogurt/water consumed until first satiation in g; total_g, total amount of yogurt/water consumed until complete fullness in g; Δfull_g, difference between total_g and sat_g; sat_%, Δfull_g divided through total_g; control, control condition; stress, stress condition; bread_g, amount of bread eaten in g. *n* = 61.

### Effects of stress induction

**Fig. 3:**
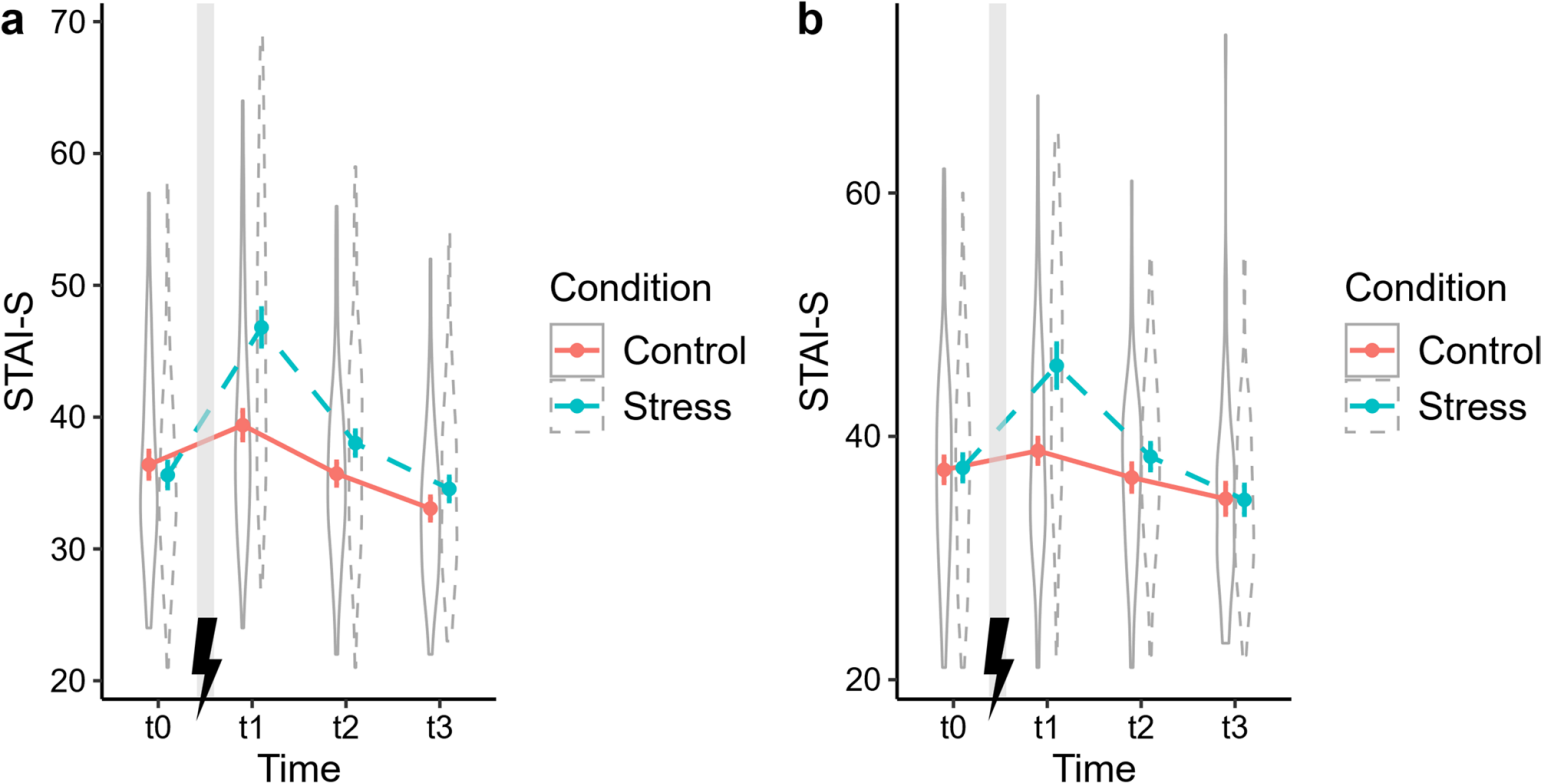
Changes in STAI-S scores over time in the stress and control conditions. Changes are shown for (**a**) the Magic Table sessions and (**b**) the two-stage Water Load Test sessions. Stress induction took place between t0 and t1. Plots showing observed means, 95% CIs, and density of STAI-S scores. Confidence intervals are based on within-subject standard errors^96^. STAI-S, State-Trait Anxiety Inventory.

The cMLMs showed that STAI-S scores changed over time (significant main effect of *Time*), and that the temporal trajectory of STAI-S scores differed between the stress and control conditions (significant *Time* × *Condition interaction)* (see Table 3 for the full cMLM summary and planned contrasts). Planned contrasts demonstrated a significant increase in STAI-scores from baseline (t0) to the first post-intervention measurement (t1) in the stress condition (MT and WLT-II sessions) and in the control condition (MT session). This increase in STAI-S scores was significantly larger in the stress condition than in the control condition (for MT and WLT-II sessions), which is reflected in the effect sizes. Figure 3 depicts the changes in STAI-S scores over time in both conditions.

**Table 3:** Results of cMLMs and contrasts for STAI-S scores.

Predictor		MT Sessions					WLT-II Session				
		df1	df2	*F*	*p*	*η*²_*p*_	df1	df2	*F*	*p*	*η*²_*p*_
Time		3	646.71	24.71	< 0.001***	0.10	3	420	6.73	< 0.001***	0.05
Time × Condition		4	646.96	29.35	< 0.001***	0.15	4	420	16.25	< 0.001***	0.13

Comparison	Time contrast	Δ	*SE*	*t*(647)	*p*	*d*	Δ	*SE*	*t*(420)	*p*	*d*
Control	t1 – t0	3.00	0.74	4.06	*< *0.001***	0.51	1.56	0.88	1.77	0.078	0.36
Stress	t1 – t0	11.20	0.74	15.24	*<* 0.001***	1.43	8.59	0.88	9.75	*< *0.001***	1.00
Stress – Control	t1 – t0	8.20	1.04	7.87	< 0.001***	1.18	7.03	1.25	5.64	< 0.001***	1.03

Δ = difference in estimated marginal means. *d* = Cohen’s *d*_z_ for within-condition contrasts and Cohen’s *d*_av_ for the interaction contrast^86^. *Time* = t0 (baseline), t1 (post-stress induction). Magic Table session: *n* = 93 (Control), *n* = 94 (Stress). WLT session: *n* = 61 per condition.

### Changes in gastric interoceptive sensitivity as a function of stress

**Fig. 4:**
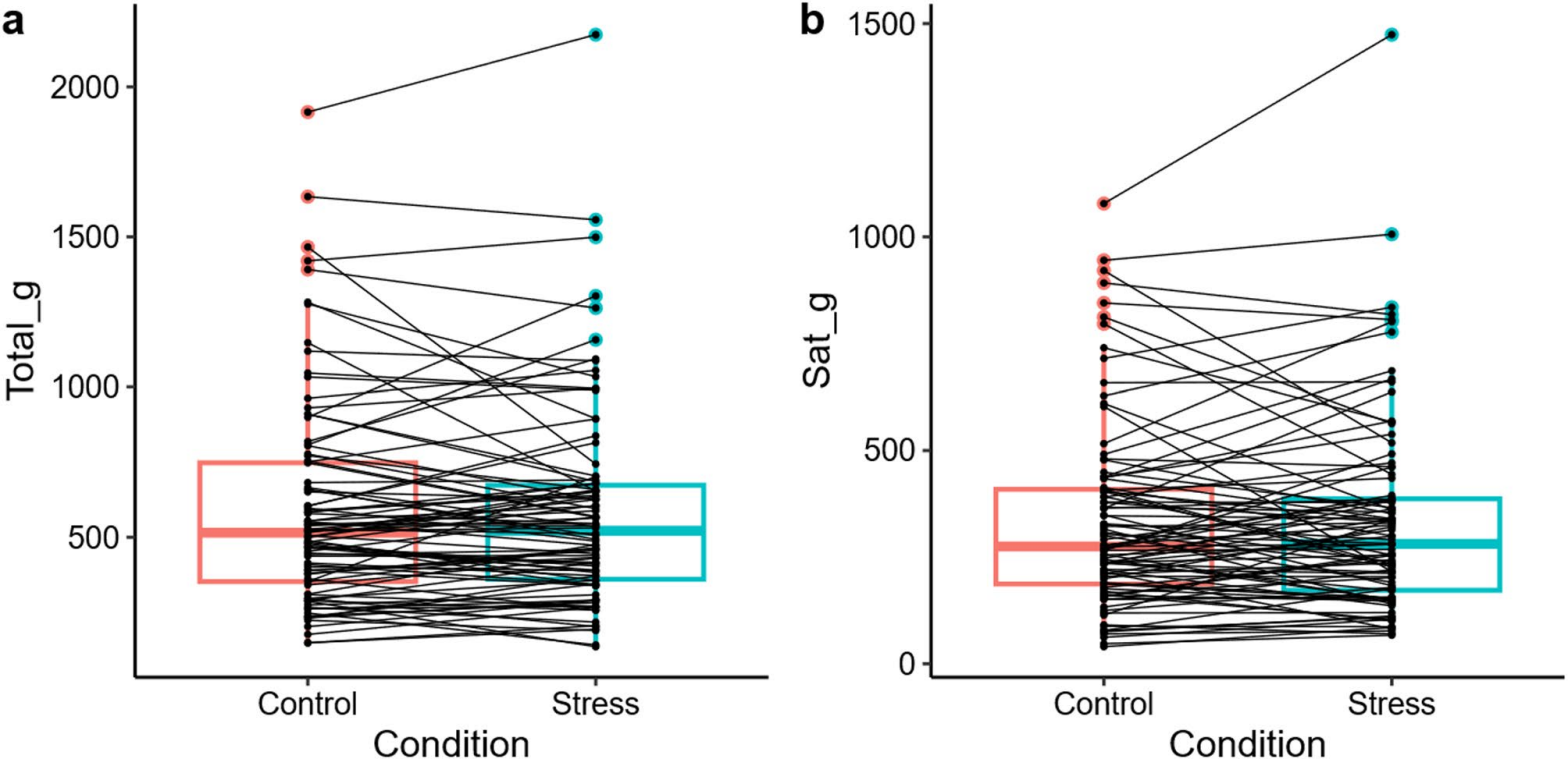
Group-level distribution and individual trajectories of gastric interoceptive sensitivity scores. Scores are shown as a function of condition for the indices (**a**) total_g and (**b**) sat_g. Connecting lines represent individual participants. The overlapping interquartile ranges of the boxplots reflect the absence of a significant main effect of condition at the group level, while the crossing trajectories illustrate substantial between-person variability in stress-induced changes. Total_g, the total amount of yogurt consumed until fullness; Sat_g, the amount of yogurt consumed until first satiation.

The MLMs did not reveal an effect of *Condition* on total_g or sat_g, respectively (Table 4). However, we observed a large between-person variability in the differences between interoceptive sensitivity in the stress and control conditions, as shown in Fig. 4. For total_g, participants ate on average 591 g (*SD* = 346) in the control condition and 586 g (*SD* = 337) of yogurt in the stress condition. For sat_g, they ate 335 g (SD = 221) on average in the control and 337 g (*SD* = 233) in the stress condition. There was a significant main effect of *Sex*, indicating that male participants had higher total_g and sat_g scores than female participants. No other main effects were found.

**Table 4:** Bootstrap results for the effect of condition (stress vs. control) on total_g and sat_g.

Fixed effects	Total_g			Sat_g		
	*b*	*SE*	95% CI	*b*	*SE*	95% CI
(Intercept)	753.50	22.62	**[710.00**,** 798.00]**	436.66	16.07	**[407.00**,** 469.00]**
BMI	26.80	26.41	[– 24.70, 80.50]	14.49	18.38	[– 21.40, 51.70]
Sex	526.05	68.46	**[396.00**,** 662.00]**	321.01	47.62	**[233.00, 417.00]**
Baseline stress	– 21.31	12.79	[– 46.60, 3.31]	– 10.77	10.15	[– 30.80, 9.14]
Stress reactivity	– 14.44	12.19	[– 39.30, 9.66]	– 5.03	10.00	[– 24.70, 14.40]
Condition	7.91	17.24	[– 26.80, 41.10]	5.12	14.50	[– 23.80, 33.10]
*R*^*2*^ conditional/ marginal	0.881/0.404			0.820/0.328		

Continuous predictors z-standardized, condition contrast-coded (control = -1/2, stress = 1/2), sex contrast coded (female = -1/2, male = 1/2). *n* = 93 (Control), *n* = 94 (Stress).

95% CIs not including zero are in bold.

#### Effect of stress moderated by emotion regulation difficulties and eating traits

**Fig. 5:**
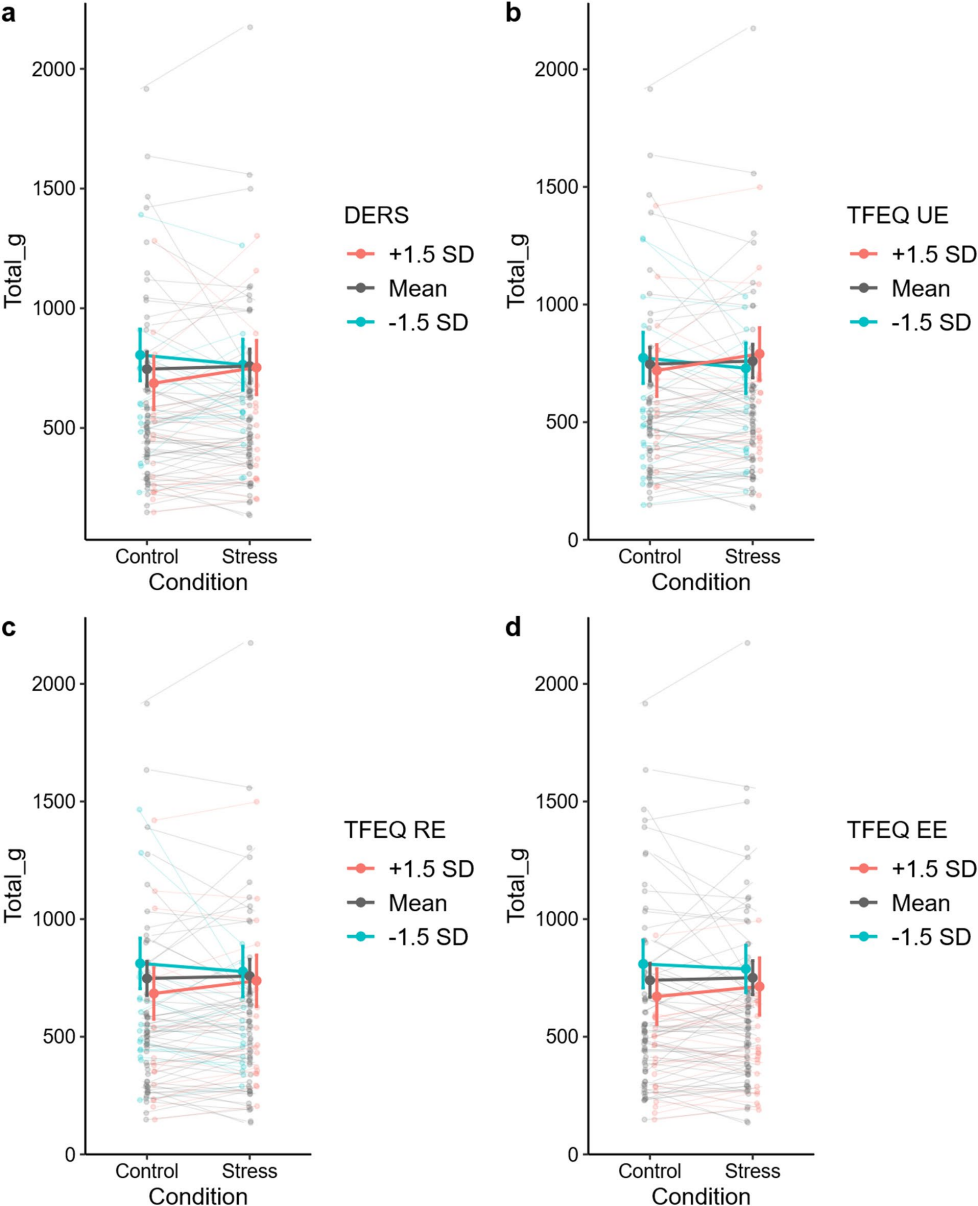
Predicted values of total_g as a function of condition and individual traits. Traits are (**a**) emotion regulation difficulties, (**b**) uncontrolled eating, (**c**) restrained eating, and (**d**) emotional eating. Total_g, the total amount of yogurt consumed until fullness. DERS, Difficulties in Emotion Regulation Scale; TFEQ RE, Three-Factor-Eating-Questionnaire restraint eating Scale; TFEQ UE, Three-Factor-Eating-Questionnaire uncontrolled eating scale; TFEQ EE, Three-Factor-Eating-Questionnaire emotional eating scale. The values for the moderators were chosen solely for illustrative purposes; in the analytical model, they were treated as continuous variables.

All model parameters are reported in Table 5. As depicted in Fig. 5(a), participants reporting higher but not lower emotion regulation difficulties, ate more until perceiving fullness in the stress compared to the control condition, indicating decreased gastric interoceptive sensitivity under stress (significant *Difficulties in Emotion Regulation* × *Condition* interaction). No *Difficulties in Emotion Regulation* × *Condition* interaction was found in predicting sat_g.

Likewise, participants with higher but not lower restraint and uncontrolled eating scores ate more under stress than under the control condition until perceiving fullness (significant *Restrained/Uncontrolled eating* × *Condition* interaction) (Fig. 5(b), (c)). For sat_g, there were no significant *Restrained/Uncontrolled Eating* × *Condition* interactions.

The emotional eating score did not explain between-person differences in changes in gastric interoceptive sensitivity thresholds in response to stress (no significant *Emotional Eating* × *Condition* interaction for sat_g and total_g) (see Fig. 5(d) for total_g).

In all MLMs, male participants had higher total_g and sat_g scores than female participants (significant main effects of *Sex*). Furthermore, in the MLM, including *Uncontrolled Eating*, we observed a significant main effect of *Baseline Stress Level*, meaning that a higher baseline stress level was related to a higher gastric interoceptive sensitivity (i.e., smaller amount eaten until complete fullness). None of the other predictors was significant in all MLMs.

**Table 5:** Bootstrap results for condition × trait moderator effects on total_g and sat_g.

Fixed effects	Total_g			Sat_g		
	*b*	*SE*	95% CI	*b*	*SE*	95% CI
(Intercept)	752.25	22.62	**[709.00**,** 798.00]**	436.41	16.16	**[406.00**,** 469.00]**
BMI	26.85	26.35	[– 24.30, 80.10]	15.52	18.46	[– 21.30, 51.70]
Sex	521.48	68.43	**[391.00**,** 657.00]**	320.11	47.89	**[231.00**,** 417.00]**
Baseline stress	– 21.22	12.94	[– 47.20, 4.37]	– 10.50	10.48	[– 31.30, 10.10]
Stress reactivity	– 19.30	12.24	[– 43.80, 5.16]	– 5.75	10.23	[– 25.90, 14.20]
DERS	– 21.56	25.88	[– 70.80, 29.90]	– 4.47	18.24	[– 39.40, 31.50]
Condition	12.42	16.99	[– 21.30, 45.00]	5.80	14.61	[– 22.90, 34.30]
DERS × Condition	35.26	12.66	**[11.10**,** 60.20]**	6.02	10.88	[– 14.90, 27.90]
*R*^*2*^conditional/ marginal	0.885/0.411			0.820/0.327		
(Intercept)	752.80	22.62	**[709.00**,** 798.00]**	436.13	16.03	**[406.00**,** 468.00]**
BMI	30.60	26.66	[– 19.70, 85.00]	17.67	18.58	[– 18.00, 55.70]
Sex	524.49	68.46	**[395.00**,** 660.00]**	319.68	47.50	**[232.00**,** 416.00]**
Baseline stress	– 22.26	12.66	[– 47.60, 2.54]	– 11.13	10.13	[– 31.20, 8.87]
Stress reactivity	– 16.04	12.03	[– 40.10, 8.16]	– 6.10	9.95	[– 25.70, 13.40]
TFEQ RE	– 27.80	25.32	[– 77.00, 22.70]	– 22.84	17.52	[– 56.50, 11.30]
Condition	9.84	16.99	[– 24.10, 42.30]	6.41	14.44	[– 22.20, 34.40]
TFEQ RE × Condition	29.51	12.65	**[5.05**,** 54.50]**	15.11	10.66	[– 5.63, 35.70]
*R*^*2*^conditional/ marginal	0.884/0.411			0.822/0.337		
(Intercept)	753.09	22.59	[709.00, 799.00]	436.59	16.13	**[406.00**,** 469.00]**
BMI	26.00	26.61	[– 25.90, 80.90]	14.53	18.59	[– 21.30, 52.60]
Sex	526.16	68.47	**[397.00**,** 653.00]**	320.96	47.82	**[232.00**,** 418.00]**
Baseline stress	– 26.40	12.71	**[– 51.80**,** – 1.91]**	– 11.34	10.38	[– 31.70, 8.91]
Stress reactivity	– 19.31	12.18	[– 44.10, 4.02]	– 5.64	10.13	[– 25.60, 14.30]
TFEQ UE	1.24	25.84	[– 47.30, 52.00]	– 1.10	17.95	[– 35.40, 34.20]
Condition	13.27	17.05	[– 20.30, 45.70]	5.81	14.61	[– 22.90, 33.50]
TFEQ UE × Condition	38.44	12.92	**[14.10**,** 64.50]**	5.18	11.07	[– 15.90, 26.80]
*R*^*2*^conditional/ marginal	0.885/0.408			0.820/0.326		
(Intercept)	745.50	23.65	**[701.00**,** 793.00]**	430.15	16.05	**[399.00**,** 462.00]**
BMI	31.19	26.72	[– 19.30, 84.10]	18.12	17.96	[– 17.30, 53.70]
Sex	501.06	71.32	**[366.00**,** 644.00]**	300.67	47.85	**[209.00**,** 397.00]**
Baseline stress	– 22.56	13.22	[– 48.90, 2.48]	– 11.29	10.47	[– 31.80, 8.73]
Stress reactivity	– 17.46	12.73	[– 42.40, 7.20]	– 7.07	10.40	[– 27.20, 13.60]
TFEQ EE	– 35.45	26.23	[– 85.90, 17.00]	– 28.94	17.77	[– 62.90, 6.74]
Condition	10.90	17.78	[– 24.10, 46.60]	7.18	14.84	[– 21.30, 36.10]
TFEQ EE × Condition	21.18	13.00	[– 4.97, 46.60]	18.29	11.07	[– 4.21, 39.40]
*R*^*2*^conditional/marginal	0.882/0.416			0.824/0.356		

Continuous predictors z-standardized, condition contrast-coded (control = -1/2, stress = 1/2), sex contrast coded (female = -1/2, male = 1/2). Total_g, the total amount of yogurt consumed until fullness; Sat_g, the amount of yogurt consumed until first satiation. BMI, Body mass index; DERS, Difficulties in Emotion Regulation Scale; TFEQ RE, Three-Factor-Eating-Questionnaire restraint eating Scale; TFEQ UE, Three-Factor-Eating-Questionnaire uncontrolled eating scale; TFEQ EE, Three-Factor-Eating-Questionnaire emotional eating scale. *n* = 93 (Control), *n* = 94 (Stress).

95% CIs not including zero are in bold.

## Discussion

The current study introduced a new method to assess gastric interoceptive sensitivity through food intake and examined its role in stress-induced eating. We observed moderate to high correlations between the gastric interoceptive sensitivity indices assessed with the new MT method and the established WLT-II, as well as with free food consumption. While no main effect of stress on gastric interoceptive sensitivity emerged, significant interactions with eating traits and emotion regulation difficulties predicted fullness but not satiation sensitivity.

For hypothesis (1), we expected positive correlations between satiation and fullness perceptions assessed by the MT and WLT-II across both stress conditions. This was largely confirmed: nearly all thresholds were highly correlated between the two methods in the same stress states, except for sat_% under high stress. Correlations for total_g and sat_g almost matched previously reported test-retest reliability for WLT-II indices^59^, supporting the criterion validity of the MT. Additionally, all MT indices (except for sat_%) showed strong correlations with free food consumption, providing initial support for the predictive validity of MT-based interoceptive sensitivity measures for eating behavior. Further research is needed to clarify the role of sat_%, which showed no link to food intake and has not previously been associated with eating or interoceptive sensibility questionnaires, nor was it shown to distinguish between individuals with and without eating disorders^45, 57^. Our findings of high correlations between sat_g and total_g and free food consumption align with prior studies validating sat_g and total_g as indices of gastric interoceptive sensitivity. These studies reported correlations with cardiac interoceptive accuracy and significant differences between eating disorder patients and healthy controls^56–58^, which collectively support the external validity of these indices. Although our correlations between the MT and WLT-II indices were high, they were not so high as to suggest interchangeability. Therefore, the MT could be used to assess whether it can capture additional variance in eating behavior beyond what is captured by the WLT-II, which focuses only on sensitivity to gastric distention. This is important because satiation during daily eating is influenced by factors such as taste, nutrient content, and hormone secretion^60^, in addition to gastric distension. Hedonic qualities (e.g., sweetness), preferences for specific tastes, and expectations based on food consistency or prior experiences may also interact with interoceptive responses^97–102^. The MT allows manipulation of these variables to examine their effects on interoceptive sensitivity. Overall, its design likely offers greater ecological validity than the WLT-II for understanding real-world eating behavior.

Hypothesis (2) concerned the effectiveness of our stress task in increasing self-reported stress. The mental arithmetic task was successful, as STAI-S scores increased significantly from baseline to post-stress, and this increase was stronger in the stress than in the control condition, both in MT and WLT-II sessions with large effect sizes^95^. These findings confirm the stress-inducing nature of the intervention, consistent with prior studies validating mental arithmetic tasks as reliable inducers of self-reported and physiological stress responses^18, 67, 103–107^. This allows the subsequent evaluation of hypotheses (3) and (4).

In hypothesis (3), we explored whether acute stress alters gastric interoceptive sensitivity. No effect of stress was found for either sensitivity index: perception of first satiation or fullness. To date, no behavioral study has evaluated how stress affects gastric interoceptive sensitivity. One self-report study suggested a link between experienced stress and reduced responsiveness to hunger/satiety signals^108^. However, prior research shows that self-reported interoceptive sensibility does not necessarily align with behavioral sensitivity measures^109, 110^. Since stress can reduce the self-reported responsiveness to hunger/satiation (as reported by^108^), but does not alter the behaviorally assessed perception of satiation, one may speculate that not gastric perception itself is impaired by stress, but rather the cognitive (e.g., attentional) resources to process gastric percepts and incorporate them into eating regulation.

These findings partly align with previous work on stress- and emotion-induced eating. While some experimental studies and a meta-analysis reported increased consumption following stress^11, 18^, others found only a small^22^ or no^21^ effect. This variability matches self-report data showing that 38–51% of individuals eat more under stress, while others eat less or show no change^24, 111, 112^. The between-person variability in gastric interoceptive responses to stress in our study may help explain these different eating patterns. Differences in stressor type or its evaluation by participants might also contribute. Stressors perceived as threats (e.g., to the social self) are linked to hypothalamic-pituitary-adrenal (HPA) axis activation and cortisol release, which increases hunger. In contrast, challenge-type stressors more strongly engage the SAM system, which suppresses digestion^113^. Public speaking tasks, for instance, elicit stronger cortisol responses than cognitive stressors like mental arithmetic tasks^114^. Thus, our arithmetic task likely resembled a challenge rather than a threat, with limited HPA activation. Future studies should include stressors that activate both systems and measure physiological markers such as cortisol. Additionally, it could be examined whether inducing specific emotions produces effects on gastric interoceptive sensitivity. Furthermore, the high intraclass correlation coefficients observed for both satiation (82.6%) and fullness thresholds (88.8%) indicate that gastric interoceptive sensitivity is largely stable within individuals across the stress and control sessions. First, this suggests that gastric interoceptive sensitivity may reflect a relatively stable trait-like characteristic, consistent with theoretical frameworks conceptualizing interoception as a trait-like ability^115^. Second, the remaining within-person variance (11.2% for fullness, 17.4% for satiation) may indicate that acute stress modulated this trait-like interoceptive index (as shown in other studies^116^) differently across individuals, even in the absence of a main effect of condition.

In hypothesis (4), we examined whether individual traits (emotion regulation difficulties, restraint, uncontrolled, and emotional eating) can explain why individuals differ in their stress-induced change in gastric interoceptive sensitivity. Results partially supported our hypothesis. None of the traits moderated stress effects on sat_g. One explanation may be that the sensation of first satiation requires the detection of a relatively weak gastric signal whose onset is difficult to identify reliably^59^. This perceptual uncertainty may induce unsystematic within-person variability in satiation thresholds (noise due to uncertainty) and thereby reduce systematic within-person changes that can be explained by our trait-moderators. Additionally, between-person differences in stress-induced changes in the satiation threshold may be driven by other psychological mechanisms than those captured by our trait moderators, which should be examined in future research.

Indeed, for fullness perception (total_g), difficulties in emotion regulation, restraint, and uncontrolled eating all moderated the stress effect (emotional eating did not). Participants scoring higher on these traits needed to eat more to feel full under stress than under control conditions. Because we used a within-subject design, each participant served as their own control, eliminating stomach capacity as a confound. One can conclude that individuals with higher scores on these traits experience reduced fullness sensitivity under high-stress compared to low-stress states. These results suggest that while the baseline capacity for gastric interoceptive sensitivity may be trait-like, its sensitivity to acute stress is shaped by between-person differences in emotion regulation and eating behavior. This aligns with previous findings that individuals high in restraint^21, 117^, and uncontrolled eating^118–120^, and emotion regulation difficulties^121, 122^ show increased food intake under stress or negative emotions. Together, these findings are in line with the idea that gastric interoception plays a role in eating regulation and that emotion regulation abilities and body-oriented eating behavior are protective factors against dysregulated eating^123^. Potential implications for the development of early prevention approaches include targeting emotion regulation skills and reducing restrained and uncontrolled eating behaviors. These components are already addressed in evidence-based treatments for bulimia nervosa and binge eating disorder^124, 125^. Based on our findings, healthy individuals with high restraint, uncontrolled eating, and emotion regulation difficulties could also benefit from interventions targeting gastric interoception in prevention programs. This warrants evaluation in future research. Our findings from a healthy sample cannot be directly generalized to clinical eating disorder populations, who represent qualitatively distinct conditions. Nevertheless, they may inform future research in this area, examining whether similar mechanisms operate in clinical populations. Such research could contribute to the development of treatment approaches. Approaches being explored in this area include interoceptive exposure adopted for eating disorders^126–128^, intuitive eating programs^129, 130^, and vagus nerve stimulation^131–134^.

The lack of a link between emotional eating and stress-induced changes in fullness perception may appear counterintuitive, given that emotional eating is defined as eating in response to emotions^135, 136^. However, many experimental and diary studies do not confirm that self-reported emotional eating predicts increased food intake under negative emotions^21, 135^. This raises questions about the construct validity of self-reported emotional eating. It may reflect beliefs or concerns about emotion-related eating rather than actual behavior, particularly in normal-weight women, who made up most of our sample^136^.

## Limitations and future directions

One limitation of this study is the young, well-educated, and predominantly female student sample, which limits generalizability. Women are at greater risk for eating pathology due to psychosocial and biological factors^137–139^, but the small male subsample precluded gender comparisons. Future studies should include more diverse populations. Our healthy sample may also display less dysregulated eating than clinical populations. However, subclinical disordered eating affects 12–22% of German adolescents and students^140, 141^, underlining the relevance of studying these patterns in non-clinical groups as well. Future studies should broaden the insights from this study by examining the mechanisms of stress and gastric interoceptive sensitivity in samples with eating disorders.

Structural gastrointestinal disorders (e.g., Crohn’s disease, ulcerative colitis) were not explicitly included as exclusion criteria, which represents a limitation of the present study. Such conditions could affect gastric distension and mechanosensory signaling and thereby gastric interoceptive sensitivity measurements^142, 143^. Future studies should implement explicit screening for gastrointestinal disorders.

Another limitation is the MT procedure’s need for an experimenter to be present during food intake, which could influence behavior through social desirability or evaluation fears. Despite minimal interaction (the experimenter pretended to be distracted by reading magazines), participants may have eaten less than they would have alone. In this context, the lack of double blinding may also have contributed to researcher bias or expectancy effects.

To ensure standardization, all sessions were conducted in the morning after fasting. However, emotional eating may be more likely in the afternoon due to lowered self-control and preference for high-calorie foods later in the day^144–146^. Thus, our findings might be more pronounced at other times of day, which should be tested in future studies.

Body mass index (BMI) was included as a covariate to account for possible differences in body composition that may influence satiation/fullness perception in the MT paradigm. BMI is a widely used anthropometric index that relates body weight to height to estimate overweight or underweight. However, it does not take into account the distribution of fat and muscle tissue, gender, or age. For example, a high muscle mass may be incorrectly classified as overweight.

Behavioral thresholds for gastric interoception inevitably reflect both individual physiological characteristics (e.g., stomach capacity or receptor sensitivity) and perception. In the present study, physiological differences among participants were controlled for by the within-subject design, in which each participant served as their own control group. Furthermore, participants were instructed to stop eating at the first perception of satiation and fullness. Deviations from this instruction, for example, due to reward-driven eating or cognitive control, may have influenced the reported thresholds, which is a typical limitation of behavioral measurements in psychology.

Finally, we did not assess cortisol responses due to the limitations of collecting saliva samples post-eating or introducing additional stressors by venipuncture. Yet cortisol has been linked to stress-related eating^12, 147–149^. Chronic stress and HPA axis activity may also play a role, as elevated baseline cortisol has been observed in individuals with binge eating disorder^150, 151^. Future studies should include cortisol and other hunger- and satiation-related hormones to better understand individual differences in stress and gastric interoception.

## Conclusion

In conclusion, our study is the first to assess stress effects on gastric interoceptive sensitivity, which is important to understand the mechanisms underlying dysregulated eating behavior and to inform future studies in eating disorder samples and on prevention programs. We introduced a new method to measure gastric interoceptive sensitivity, the MT, which was designed to further develop the previous WLT-II. Our findings provide support for the validity of the MT, as its measures of gastric interoceptive sensitivity positively correlated with those of the WLT-II and free food consumption. Furthermore, our findings suggest that stress modulates gastric interoceptive sensitivity depending on eating behavior traits and emotion regulation: We found a reduction in the sensitivity to perceive fullness in individuals with high but not low emotion regulation difficulties and restrained and uncontrolled eating traits under high-stress compared to low-stress states. Hence, gastric interoceptive sensitivity may be a relevant mechanism in the regulation of eating behavior in response to stress. These results may inform future studies on similar mechanisms in eating disorders, as well as studies examining the effectiveness of interventions targeting gastric interoceptive sensitivity to prevent dysregulated eating.

## Supplementary Information

Below is the link to the electronic supplementary material.

Supplementary Material 1

## Data Availability

The dataset analysed during the current study is available in the Open Science Framework repository at https://osf.io/7ywpj/.
