# Supplementary material for: Stress modulates gastric interoception depending on eating traits and emotion regulation: evidence from the magic table
Source: Sci Rep. 2026 May 13;16:14969. doi: 10.1038/s41598-026-48641-w (PMC13171930; doi:10.1038/s41598-026-48641-w)
Supplement: Supplementary file 1 — Supplementary Material 1 [file 41598_2026_48641_MOESM1_ESM.pdf]

# Supplementary Information for: Stress modulates gastric interoception depending on eating traits and emotion regulation: Evidence from the Magic Table

Kipping, Miriam; Schulz, André; Pollatos, Olga

## The Magic Table

The equipment of the Magic Table (MT) consists of a table with an opening in the center. A scale is built into the space beneath this opening (Figure 1a). A custom-fitted bowl can be placed into the opening so that it rests precisely on the scale (Figure 1b). The bowl has a small opening on the bottom. This opening can be connected to a syringe using a food-safe tube (Figure 2). The syringe is filled with a thick liquid food (in our case, yogurt). It is then inserted into the homemade setup inside the table and connected via the tubing to the recessed bowl (Figure 3). The bowl is then also filled with 500 g of yogurt. Additional syringes filled with the food are placed in the table. In preparation for the experiment, each syringe is weighed in its filled state.

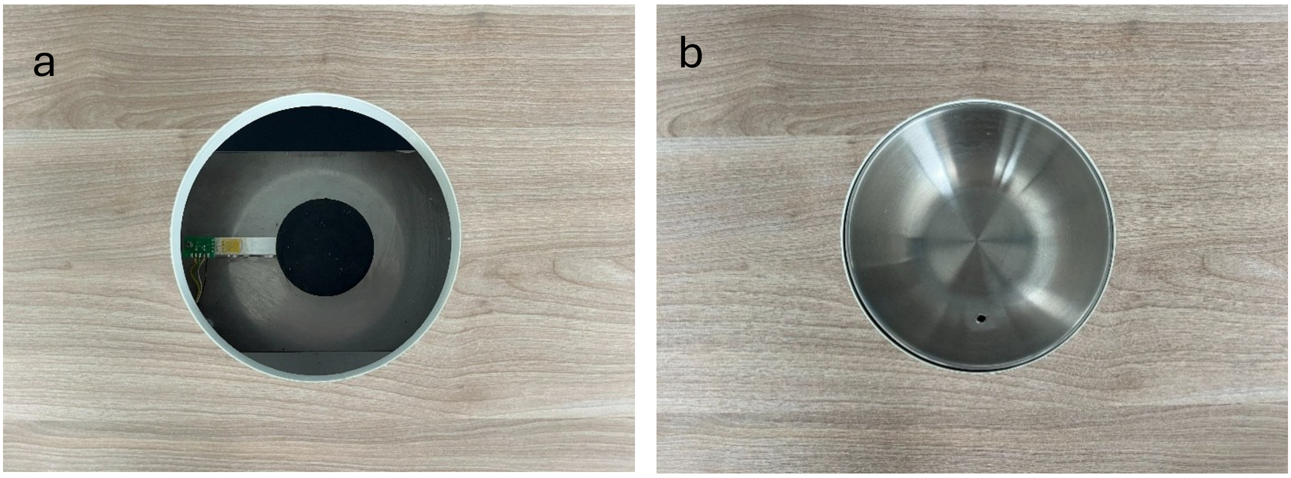


**Figure 1 | Magic Table scale and bowl.** (a) Depiction of the opening in the table with the scale built inside. (b) The Opening in the table with the custom-fitted bowl inside resting precisely on the scale.

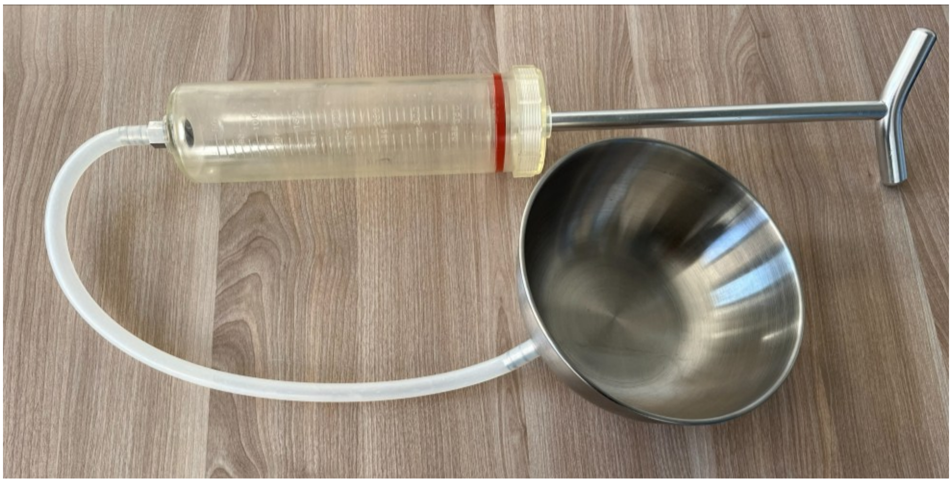

**Figure 2 | Connection of bowl and syringe with food-save tube.**

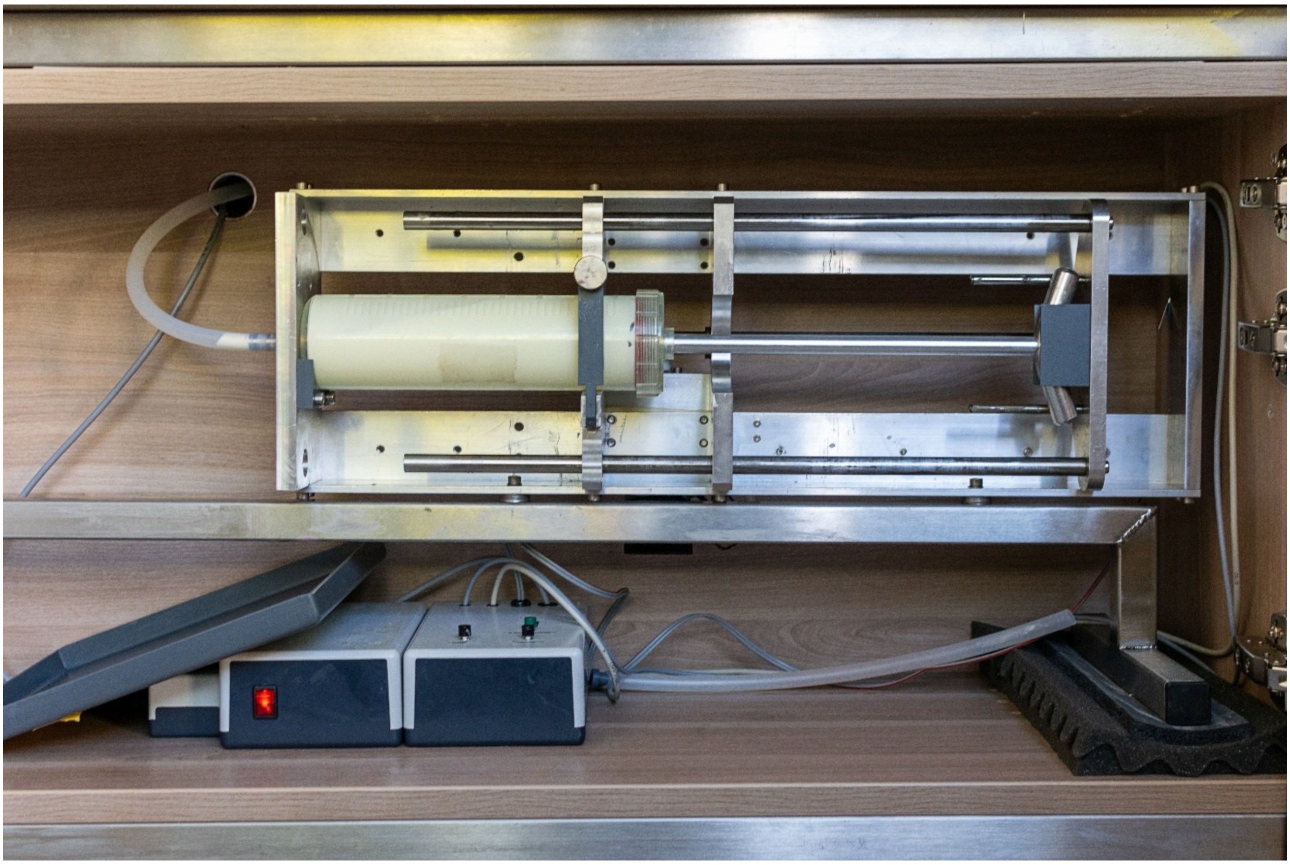

**Figure 3 | Homemade setup inside the table.** The syringe can be inserted into the setup within the table. The automatic refill system can be activated using a button in the table. Then the scale measures the current weight of the bowl. A motor in the table then empties the content of the syringe, keeping the weight of the bowl – and thus the amount of food – constant.

To measure the satiation thresholds, the participants sits down at the table. They see only the filled bowl, not the syringes or the construction inside the table (Figure 4). To start the experiment, the experimenter presses a button inside the table, thereby initiating the automatic refilling of the bowl. The mechanism works as follows: As soon as the button is pressed, the scale measures the weight in the bowl (here 500 g, before the participant starts to eat). If the participant begins to empty the bowl while eating, causing the bowl to become lighter, the setup inside the table empties the syringe in such a quantity that the bowl is constantly refilled to its initial weight (500 g).

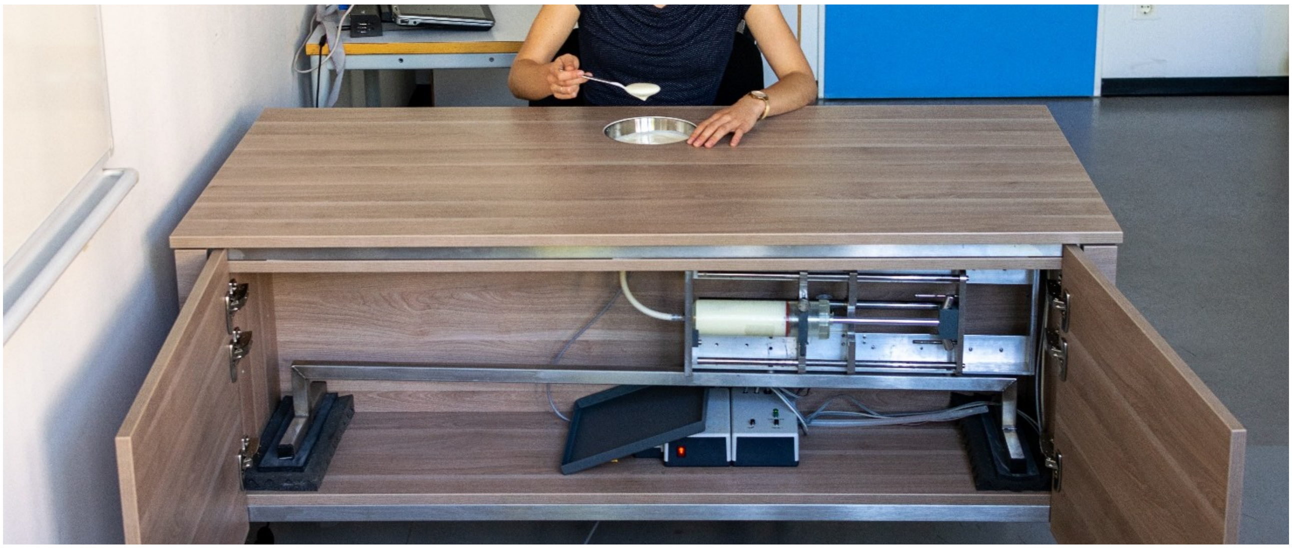

**Figure 4 | Participant sitting at the Magic Table.** While participants are eating a hidden scale under the bowl and a hidden syringe and pump inside the table maintain a constant amount of yogurt inside the bowl.

The experimenter sits at a 90-degree angle to the participant at another table in front of them, does not watch the participant, and pretends to be distracted by something else. Out of the corner of their eye, they must watch the inside of the table to see if the contents of the syringe are running low. If this is the case, they ask the participant to pause eating briefly so they can "adjust something." They wait until the table has refilled the bowl to its original level. Then they replace the nearly empty syringe with a full one. The participant cannot see this, as everything takes place inside the table. Once the syringe has been replaced, the experimenter presses the button again to initiate the automatic refilling of the bowl. They then inform the participant that they may continue eating. When the participant indicates that they have reached the first satiation threshold, they are also asked to pause eating briefly. The current syringe is replaced again with a full one ("I need to adjust something in the table"). This is followed by the instruction to eat until the second satiety threshold is reached. Here, too, the experimenter monitors the syringe's fill level and replaces it should the contents run low (as described above).

After the subject has left the room at the end of the experiment, all syringes used are weighed again, and the amount of food consumed is calculated as the difference between the initial weight and the current weight.
